# What is a capable guardian to older fraud victims? Comparison of younger and older victims’ characteristics of online fraud utilizing routine activity theory

**DOI:** 10.3389/fpsyg.2023.1118741

**Published:** 2023-06-07

**Authors:** Katalin Parti

**Affiliations:** Cybercriminology Lab, Department of Sociology, Virginia Tech, Blacksburg, VA, United States

**Keywords:** cyber routines, capable guardian, older adults, victimization, online fraud, routine activities theory, scam

## Abstract

**Objective:**

The paper compares victim group characteristics: we test routine activities theory to compare the differences in online fraud vulnerabilities of victims aged 18–54 and victims of 55 and above.

**Methods/sample:**

A representative sample of US citizens 18 and above was collected in October 2020. Victims under 55 encompassed 35.3% (*n* = 915), victims 55 and above 12.9% (*n* = 334) of the total sample (*n* = 2,589). We utilized non-parametric statistical methods for testing whether older and younger victims’ characteristics can be derived from the same independent variables.

**Results:**

Computer time, computer familiarity, and technical guardians determine online victimization in older individuals, similarly to younger age groups. However, older victims differ in characteristics from younger victims. Seniors were less likely to apply technical guardians such as camera cover, identity theft monitoring, and credit card freeze, even after experiencing online scams. Being a single parent was a protective factor for older individuals, but having a full-time job made older individuals more prone to experience online fraud victimization compared to being retired. In addition, older victims were less likely to report scams than younger ones.

**Conclusion/implications:**

Although this research found significant differences between older and younger fraud victims’ characteristics, target suitability and capable guardianship must be further investigated and conceptualized when applying routine activities theory for online fraud against older people.

## 1. Introduction

Older individuals become increasingly vulnerable to online fraud with their daily routines migrating to online platforms. Burnes et al. (2017), in a meta-analysis of 12 studies involving 41,711 individuals, found that online fraud affects approximately one in every 18 cognitively intact, community-dwelling older adults a year. The overall prevalence of elder financial fraud across studies was 5.6% up to 5 years and 5.4% up to 1 year. Financial fraud victimization is associated with severe financial, physical, and mental health consequences, including major depression, generalized anxiety disorder, lower subjective health ratings, increased somatic complaints (Ganzini et al., 1990), premature mortality, and greater hospitalization (Dong and Simon, 2013; Burnett et al., 2016). In addition, most fraud-scam victims report anger, stress, regret, embarrassment, sadness, helplessness, and shame (FINRA, 2016). Older adults lost an estimated $1 billion (IC3, 2020) to online fraud in an age when loss recovery is difficult, and financial savings would be necessary to cover health insurance. Burnes et al. (2017) estimate that elder fraud/scam cases will double over the next two or three decades due to older adults’ population growth.

In this study, we define online financial fraud/scam as intentionally deceiving a victim by misrepresenting, concealing, or omitting facts about promised goods, services, or other -- physical, mental, or emotional -- benefits that are nonexistent, unnecessary, or deliberately distorted for monetary gain (adapted from Titus et al., 1995; Beals et al., 2015b). Financial fraud differs from financial exploitation/abuse that is committed by caregivers or other trusted individuals (Hall et al., 2016). Our knowledge about elder financial fraud is mainly limited to elder financial abuse. However, little is known about seniors’ specific situational and sociodemographic risks of falling for financial fraud/scams (Burnes et al., 2017). Our study aims to fill that gap.

Despite its severe effects and prevalence, fraud targeting older adults has not yet been studied extensively from a social context point of view, partly because of the inaccessibility of older age groups. In addition, among the works that study financial fraud victimization of older individuals, financial exploitation fraud by caretakers and financial fraud by strangers are often studied together, resulting in general findings. Furthermore, these studies do not include cases where perpetrators only interacted with victims online or by phone (Burnes et al., 2017; DeLiema, 2018). On top of that, the relationship between social context variables (demographics, living arrangements, job market activity) has not been revealed. This is a consequence of the lack of available data on the sociodemographic characteristics of the victims (DeLiema, 2018; Whitty, 2019). Thus, the current study focuses on victim demographics and situational circumstances, revealing unique findings of risk and protective factors for older victims, compared to younger victims, utilizing routine activity theory.

## 2. Theoretical framework: routine activities theory

Routine activities theory (RAT; Cohen and Felson, 1979) suggests that an individual’s daily activities contribute to victimization. Cohen and Felson (1979) posit that an individual’s social roles and social class influence their lifestyle, including risky activities resulting from individual rational choice. They suggest that a crime will likely occur if (1) a suitable target, (2) a motivated offender, and (3) the absence of a capable guardian spatio-temporarily converge. All three must come together in order for criminal activity to be realized. However, according to Cohen and Felson (1979), the motivated offender is everywhere, awaiting the opportunity to engage in criminal activity. Thus, research should concentrate on the victims’ routines instead.

RAT has been initially developed to study victimization of predatory and property crimes (Cohen and Felson, 1979; Felson, 1986 Miethe et al., 1987; Massey et al., 1989; Sherman et al., 1989; Kennedy and Forde, 1990; Roncek and Maier, 1991), Newman and Clarke (2003) applied the model to cyberspace. They argued that online presence alone could make people suitable targets since they publish personal information as valuable assets online. Subsequent research found evidence for RAT in computer-crime (Kowalski, 2002; Moitra, 2005; Choi, 2008) and internet-crime victimization (Choi and Lee, 2017; for a summary see Leukfeldt and Yar, 2016). These studies posit that cyberspace provides ideal opportunities to commit crimes, as targets are digitally connected with multiple devices, working, studying, networking, and gaming online. Thus, all who are digitally connected suitable targets of online crimes. In the integrated cyber-RAT Choi (2008) asserts that motivated cybercriminals can easily find suitable targets in the form of online users who engage in online activities without applying adequate computer security measures or sharing their data on social networks (Yar, 2005). Computer security measures such as anti-virus software, web camera cover, or credit card monitoring function as capable (technical) guardians, and absence of them increases offending opportunities (Leukfeldt and Yar, 2016; Hawdon et al., 2020). At the same time, the level computer familiarity and computer skills can affect target suitability (Bossler and Holt, 2009). For instance, Choi (2008) tested target suitability studying risky online routine activities such as user willingness to visit unknown websites, downloading free MP3 files or free software programs, and clicking on icons without precaution on a sample of college students (*n* = 204). The study measured digital capable guardianship through the application of antivirus programs, antispyware, and firewall programs. Online lifestyle variables, such as online vocational and leisure activities, online risky leisure activities, and online risky vocational activities, have been used to measure the suitable target component. Choi (2008) concludes that online lifestyle activities contribute to the potential for computer crime victimization.

The current study proposes an extension of the measures of capable guardianship and target suitability to measure RAT applicability in financial fraud. Specifically, we propose that the presence of social (capable) guardians, such as relatives, can provide protection against financial fraud/scams targeting older individuals. We also suggest that victims’ sociodemographic characteristics – such as education level, employment status, and asking for help after the initial fraud victimization – also influence target suitability. By including these new measures in the analysis, we suggest extending the list of measures in capable guardianship and suitable target, therefore, updating RAT measures to study financial scam victimization. In addition, we propose age-dependent characteristics of these measures presuming they work differently for older and younger age groups. In the following, we review scientific literature in RAT and older individuals’ financial fraud victimization. Then, we describe the sample and methods applied in the current study, present the results, and discuss the findings of the current study considering the literature. The paper concludes with policy and future research recommendations.

## 3. Literature review. Older people’s target suitability and the lack of capable guardians

Although older adults’ overall crime victimization is lower than that of the younger ones (Carcach et al., 2001; Graycar and James, 2001; Holtfreter et al., 2014), out of the crime that older people experience, fraud is the largest category (Temple, 2007; Smith and Budd, 2009). Gamble et al. (2014) associate financial fraud victimization of older Americans with decreasing cognition, overconfidence in one’s financial knowledge, and willingness to take financial risks relative to non-victims. But even among older adults without cognitive impairment, age-related changes in cognition are associated with the declining ability of decision-making, thus, greater susceptibility to financial fraud (Boyle et al., 2012). Holtfreter et al. (2014) conducted telephone interviews on consumer fraud with 2,000 Arizonians and Floridians over 60. Fraud victimization was relatively low, with approximately 14% past-year prevalence. Being male, engaging in remote shopping, having low self-control (impulsivity), having a higher level of education, and telemarketing purchases increased fraud *targeting* (attempt to defraud the individual); remote shopping/purchasing, low self-control, being older, and minority status increased (actual) fraud *victimization*.

Older adults can downplay cognitive deficits to maintain financial independence (Deevy and Beals, 2013). Many victims never report their victimization and even hide it from family members and caretakers for fear of being blamed (Cross, 2016). That results in capable guardians (i.e., relatives, family members) not being able to step in before a greater amount of financial loss manifests. It also results in underreporting of financial fraud victimization relative to other age groups (Beals et al., 2015a). Underreporting not only distorts data on fraud victimization (Burnes et al., 2017) and limits our understanding of older people’s fraud victimization but also hinders the development of prevention programs and policies focusing on age-appropriate needs (DeLiema, 2018).

Few studies have tested RAT on older people’s cyber fraud victimization. Hutchings and Heyes (2009) concluded that computer use predicted receiving a phishing email. Reisig and Holtfreter (2013) tested the theory on remote purchasing fraud among adults 60 or older. They found that older adults who engage in remote purchasing activities face a greater risk of being targeted by fraud. Lower self-control also increased the risk of fraud victimization (Holtfreter et al., 2008; Reisig and Holtfreter, 2013). Pratt et al. (2010) examined the influence of routine online activities on Internet fraud targeting. Before controlling for time spent online and online shopping/purchasing behavior, younger and more educated consumers were significantly more likely to experience Internet fraud targeting (i.e., the attempt to defraud). But these effects disappeared after controlling for time spent online and online purchasing, and both of these behaviors significantly increased the odds of Internet fraud targeting, a finding that lends support to routine activities theory.

Although situational factors have been examined, few studies have indicated the significance of sociodemographic information in impacting or determining online fraud victimization of older adults. DeLiema (2018) adapted RAT to older individuals’ financial fraud victimization. The model suggests that aging individuals will be most vulnerable to fraud during the period of declining cognitive and physical functioning when these deficits are hardest to recognize by capable guardians such as family members or medical professionals (DeLiema, 2018, p. 708). The American Association of Retired Persons surveyed 745 telemarketing fraud victims over 50 in 1995 to develop a profile (AARP, 1996). Although victims were generally socially integrated, they were more likely to live alone than their age group of older Americans in general and also less likely to seek advice on financial matters than non-victims. Social isolation is considered to be a risk factor in financial fraud (Fenge and Lee, 2018) because it is associated with loneliness, as isolated persons can be more receptive to answering the calls of unknown telemarketing callers (Langenderfer and Shimp, 2001), doorstep sales, and scam mails, and listen to sales pitches (Lee and Geistfeld, 1999) simply for the opportunity to talk to someone.

Furthermore, the proxy of caring relatives can provide external control. DeLiema (2018) extracted data from 53 fraud and financial exploitation cases, drawn from a pool of 924 cases presented between 2006 and 2013. She found that significantly more fraud victims – victimized by predatory strangers – were childless and had no relatives nearby compared to financial exploitation victims – victimized by trusted family members and relatives. Fraud perpetrators took advantage of elders when they had no trusted relatives or friends to safeguard their assets. On the other hand, elder fraud victimization occurred most likely when the victim only had mild cognitive or physical functioning impairment, and their behavior had not yet alerted relatives and friends to step up as capable guardians. Pratt et al. (2010) found that demographic characteristics such as gender, age, education, and marital status shape routine online activities, and indicators of routine activities, such as hours spent online, and online purchasing activity mediate the effect of demographic characteristics on the likelihood of being targeted by cyber fraud. In another study, Reyns (2015) examined whether online activities increased the risk of online victimization. Individuals active in online shopping, social networking, and posting information online were more likely to be victimized by phishing, hacking, and malware. Surprisingly, online technical guardianship was negatively correlated with online victimization: those who installed antivirus software were more likely to become victims. However, victims might have installed the software after being victimized. Therefore, Reyns (2015) suggests further studying online activities, guardianship, and victimization.

Whitty (2019) developed a susceptibility theoretical framework by including personality and sociodemographic characteristics, exposing online activities, risky online spaces, and online guardianship behaviors. One explanation for why educated people are more likely to be scammed is that highly educated people have a more robust online presence and frequent more online spaces than lower-educated people. Another possible explanation derives from the work of Lea et al. (2009), who suggest that overconfidence in recognizing scams places people at greater risk of becoming scammed. Educated people might have a false sense of security and spend less effort finding manipulation cues. Whitty (2019) also found that online guardianship behaviors, such as seeking advice on fraud information sites (Federal Bureau of Investigation, Federal Trade Commission), did not protect people from being victimized. Overall, research findings highlight the need for further studying the connection between online routines, guardianship, and online victimization.

Having a job means financial security, but it also means more financial assets to lose. The COVID-19 pandemic led to significant job losses. In the meantime, computer-assisted and online work became the norm instead of an exception. Individuals’ online presence increased because of public health-related lockdowns and remote working conditions. So far, a few studies have examined the connection between lockdowns and online victimization (Hawdon et al., 2020). Kennedy et al. (2021) surveyed over 2,200 American adults to investigate their experiences with COVID-19-related fraud. People who considered themselves targets of fraud were almost 10 years younger, significantly more likely to be male, non-White, possess a Master’s or Doctoral degree, be single/never married, be employed and have a higher salary. Although the study did not look at the age differences of targeted/victimized persons, it highlights the risk factors posed by having a full-time job and living alone, suggesting that employment and the absence of family members or relatives as informal capable guardians increase the stakes of experiencing a financial loss (see also Kennedy et al., 2021).

In the present study, we have looked at situational and demographic characteristics of victims to investigate what makes older adults suitable targets for online scams and the potential protective factors, such as capable technical and nontechnical guardians. Considering previous research findings, we hypothesized that older victims differ in characteristics from younger victims. We break down these characteristics as follows:

*H1 – Older individuals who are more bound to the computer in their daily routines and more familiar with the computer (overconfidence) will be more likely victims of online fraud than younger adults with similar suitable target characteristics.*

*H2 – The presence of trusted relatives as social capable guardians protects older adults from online fraud*. Thus, those older individuals who live alone, without a partner or grown-up children, are more likely to be victimized than those who live in different living arrangements since the presence of relatives/family members can function as capable guardians.

*H3 – Older victims are less likely to report and ask for help than younger victims*. We conceptualize reporting or asking for help as a form of gaining knowledge about financial scams, which could potentially lower the level of target suitability by successfully avoiding scams in the future.

*H4 – Having a full-time job makes older people more prone to online victimization than retired individuals*. We assumed that full-time jobs provide more computer access than part-time jobs and unemployment.

In the next section, we introduce the sample, describe the variables utilized to measure suitable targets and capable guardians, introduce and discuss the results by comparing younger and older victims’ characteristics and risk factors of falling victim to typical online fraudulent scenarios. Finally, we interpret the results in light of previous research. Finally, we conclude with policy and research recommendations.

## 4. Methodology

### 4.1. Analytical strategy

**Figure 1:**
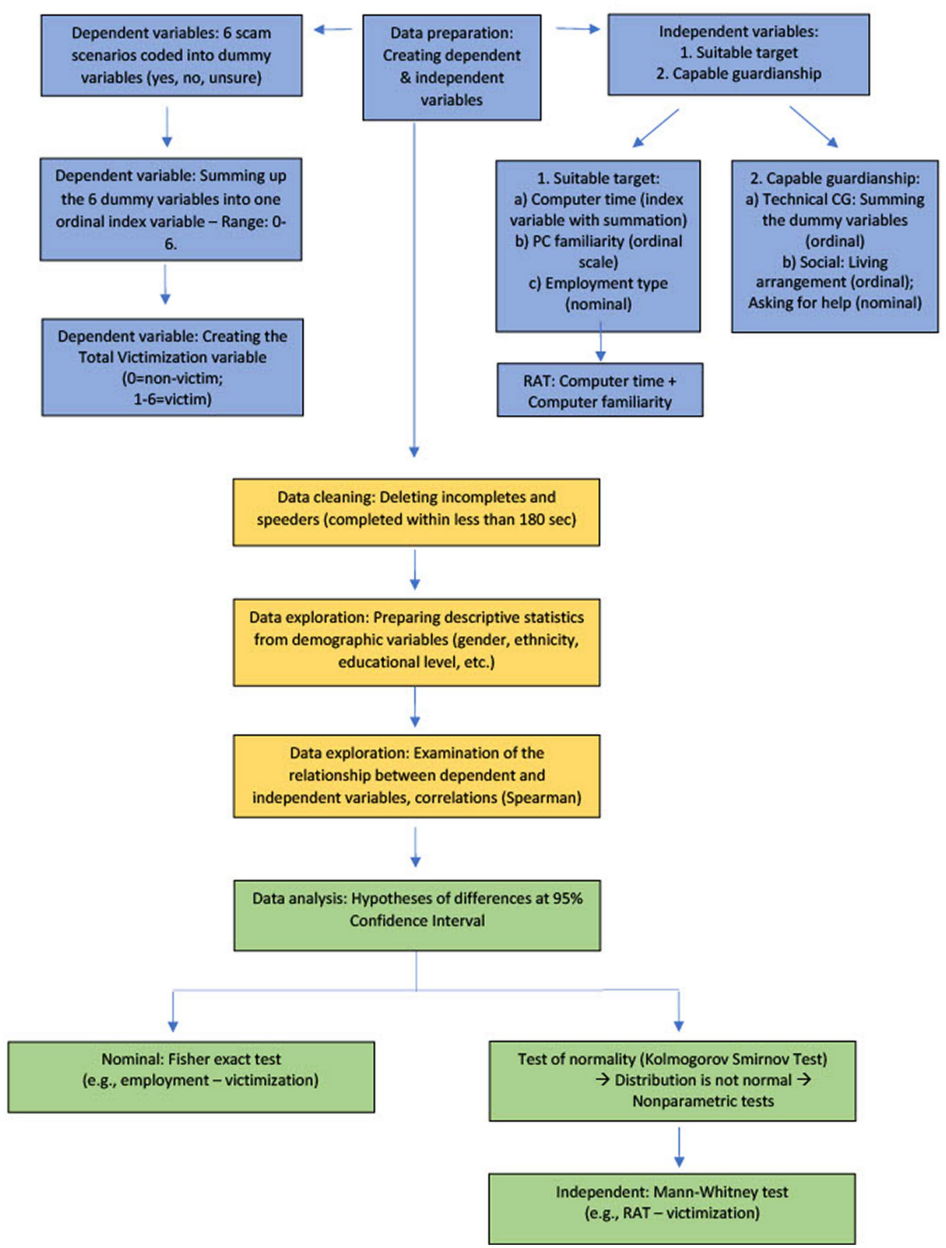
Analytical strategy flowchart.

After examining the relationships between the dependent and independent variables, Fisher’s Exact test was used because there were two dichotomous variables in our crosstabulations, e.g., whether the respondent used email (yes = 1; no = 0) and the victim was young (0) or old (1), and there were too few items in the subsamples. The Kolmogorov–Smirnov test of normality was utilized to assess the distribution level because the sample was greater than 2,000 (Lopes, 2011). Since the Kolmogorov–Smirnov test proved that the distribution of variables was not normal, non-parametric tests followed. As such, Mann–Whitney U tests were performed to compare younger and older participants’ victimization in relation to routine activities theory (Figure 1). Mann–Whitney U tests are frequently used in clinical trials (Hart, 2001; Tai et al., 2022), as an alternative to a *t* test when the data are not normally distributed (i.e., skewed), as was in our sample. Aligning with the requirements (Nachar, 2008), we applied non-parametric tests, statistical methods based on signs and ranks, for multiple reasons: (1) the combined distribution of the variables was not normal; (2) where subsamples were compared (young victims, older victims, young non-victims, older non-victims); and (3) the low number of items did not allow the use of parametric tests. In addition, (4) the measurement level suggested utilizing non-parametric tests since parametric procedures cannot be applied to ordinal variables where the mean ratio cannot be interpreted (Kitchen, 2009; Nahm, 2016). To further analyze the differences between younger and older age groups along with victimization and non-victimization (four different groups, Table 1), we utilized Kruskal-Wallis H tests which can be applied when the variable of interest is not normally distributed and when there are three or more groups in the analysis (Bewick et al., 2004; Ostertagova et al., 2014). Thus, utilizing the SPSS 27 software package, we applied non-parametric tests such as the Mann–Whitney U test instead of two-sample t-tests, the Kruskal-Wallis H test instead of one-way ANOVA, and Spearman correlations for variables with ordinal level of measurement instead of Pearson correlation (Analytical strategy flowchart shown in Figure 1).

**Table 1:** Computer familiarity, PC time, and technical guardians, by victim and non-victim age groups.

RAT variables and victim age groups – Kruskal-Wallis test						
	Mean Ranks				Kruskal-Wallis H	*p*
	Victim group (18–54)	Victim group (55+)	Non-victim group (18–54)	Non-victim group (55+)		
Computer familiarity (How familiar would you say you are with computers?)	1410.47	1169.2	1306.97	1062.64	84.666	0.001
Technical Guardian – Currently in place	1499.13	1251.6	1034.44	1127.2	200.834	0.001
Technical Guardian – Put in place because of a prior victimization	1624.94	1171.51	1081.1	1039.83	461.785	0.001
RAT Scale (PC-time + Familiarity)	1270.19	1081.59	1192.42	934.44	83.535	0.001
Technical Guardian - Pairwise comparisons with Mann–Whitney test						
		Mean Ranks		Mann–Whitney U-stat	*p*	
*Technical Guardian - Currently in place*						
Victim group (55+) vs. Victim group (18–54)		513.71	644.45	114,910	*p* < 0.01	
Victim group (55+) vs. Non-victim group (55+)		432.03	388.14	70774.5	*p* < 0.05	
Victim group (55+) vs. Non-victim group (18–54)		636.85	531.6	106,473	*p* < 0.01	
*Technical Guardian - Put in place because of a prior victimization*						
Victim group (55+) vs. Victim group (18–54)		458.77	685.68	97285.5	*p* < 0.01	
Victim group (55+) vs. Non-victim group (55+)		440.04	395.48	73,299	*p* < 0.01	
Victim group (55+) vs. Non-victim group (18–54)		607.69	563.04	125,853	*p* < 0.05	

### 4.2. Participants

A national sample of 18 or older Americans (*n* = 2,928) was collected using Dynata and Qualtrics research panels in October 2020. Dynata and Qualtrics are online market research companies offering to sample and field online surveys on samples representative of age, gender, race, and ethnicity. After deleting participants who sped through or failed to complete the survey, 2,589 items remained in the final sample. The mean age was 44.68 (*SD* = 17.35), ranging from 18 to 98 years. Overall, 48% of the sample was male, 50% female, and 0.7% non-binary, with 0.6% not answering. Regarding the highest level of education, 20% owned a postgraduate degree, 29% had a college degree, 23% some college, 23% high school, and 3% less than a high school diploma. The majority (74%) of the participants were White, 15% were Black, and 15% were of another race. Eight percent were still in school, 40% had a full-time job, 16% had a part-time job, 21% were unemployed, and 21% were retired. Twenty percent of the participants lived alone, 30% lived with a partner without children, another 26% with a partner and children, 7% were single parents, 9% were living with parents, and 7% lived together within other family types or other arrangements. Participant sociodemographic information can be found in Table 2.

**Table 2:** Total sample (*N* = 2,589) demographics.

Gender	Male	Female	LGBTQ/Non-Binary	No Answer			
	1,242 (48%)	1,295 (50%)	18 (0.7%)	15 (0.6%)			
Education	Less than High School	High School	Some College	College Degree	MA/Professional/PhD		
	73 (3%)	606 (23%)	605 (23%)	759 (29%)	527 (20%)		
Race	White	Black	American Indian	Asian	Pacific Islander /Hawaiian	Other/Prefer not to Answer	
	1905 (74%)	386 (15%)	74 (3%)	151 (6%)	18 (1%)	128 (5%)	
Age	Mean	Median	SD	Min	Max		
	44.68	42	17.35	18	98		
Employment	In school	Full-time job	Part-time job	Unemployed	Retired		
	204 (8%)	1,047 (40%)	415 (16%)	552 (21%)	535 (21%)		
RAT – PC time	Mean	Median	SD	Min	Max		
	28.48	20	26.24	0	246		
RAT – Computer familiarity	Uncomfortable	Surf the net	Fix some problems	Fix most of the problems	Programming		
	260 (10%)	733 (28%)	744 (29%)	446 (17%)	1,392 (15%)		
RAT – Technical Guardian	Cover the web camera 1,162 (44.9%)	Identity theft protection 1,118 (43%)	Freeze credit card 761 (29%)	Antivirus/Firewall 1,649 (64%)			
RAT – Living arrangement	Living alone 512 (20%)	Living w partner, no children 787 (30%)	Living w partner and children 672 (26%)	Single parent 189 (7%)	Living with parents 236 (9%)	Other family type 147 (6%)	Other arrangements 25 (1%)

### 4.3. Dependent variables

#### 4.3.1. Online fraud types

We created multiple closed-ended questions describing specific fraud-scam events to measure financial fraud affectedness in the survey. This closed-ended and descriptive assessment leaves less room for participant confusion and improves the identification of financial fraud scenarios by cueing-activating memory recall (Deevy and Beals, 2013; Burnes et al., 2017). Using the Federal Trade Commission’s fraud-scam taxonomy targeting older adults (Simons et al., 2020) and the FBI’s “elder fraud” list (FBI, 2020) as baselines to determine typical fraud scenarios targeting older Americans, we included six financial fraud scenarios, contextualizing with descriptive content: (1) Company impersonation: “In the past 12 months, did you get a phone call from a company that asked you to go to your computer and send them private information about yourself and/or your family members, and/or send them money?”; (2) Tech support scam: “Some scammers call people pretending they are from an IT company or personnel, asking to allow remote access to the computer, and once they are given access, they lock the owner out. Then they ask for credit card details to repair the owner’s computer. In the past 12 months, did you experience the above scenario?”; (3) Grandparent scam: “Some scammers call people pretending they are their grandchildren, asking for money to solve some unexpected financial problem (overdue rent, payment for car repairs, bail, etc.). At the same time, the caller begs, “please do not tell my parents.” In the past 12 months, have you received a call from such a scammer?”; (4) Personal data verification scheme: “In the past 12 months, have you received email from a seemingly legitimate company or institution (e.g., IRS, bank, etc.) asking you to “update” or “verify” your personal information via email or on the website provided by the email?”; (5) Advance fee fraud: “In the past 12 months, have you received a call or email according to which you are required to send money to someone so that at the end of the cycle (when everyone pays a certain amount of dollars) you receive a greater amount of money?”; (6) Romance scam: “In the past 12 months, have you been asked by someone you met on an online dating platform to send them money or other donations (e.g., plain tickets, travel expenses, etc.) or finances (e.g., pay for surgery or other medical expenses, pay custom fees to retrieve something, pay off gambling debts, pay for visa or other official travel documents, reload cards or gift cards)?” The answer options for all six fraud scenarios were yes (1), no (0), and unsure. “Unsure” answers were later recoded as missing values for ambiguity and to be able to dichotomize variables. An additional screening question asked whether participants were financially victimized because of being *targeted* by scammers. Only those who answered “yes” were included in the analysis.

### 4.4. Independent variables

#### 4.4.1. Suitable target

Several variables representing suitable target and capable guardian RAT measures were included in the survey. Suitable target measures were conceptualized by computer time, computer familiarity, and employment type. Computer (PC) time (computer-related activity) was measured with a time scale (hours): “In a typical week, how many hours do you spend:” “Playing video games;” “Reading news or other articles online;” “Browsing social media like Facebook, Instagram, Twitter, etc.;” “On a computer, while working at a job;” “Shopping online;” and “Other online activities.” Computer familiarity was assessed by a five-point ordinal scale ranging from “I am uncomfortable using a computer;” to “I am comfortable manipulating or writing computer programming.” Employment type was a nominal scale with the options “in school,” “paid full-time job,” “part-time job,” “unemployed,” and “retired.” Respondents could mark one of these options that best describe their employment status.

#### 4.4.2. Capable guardianship

Capable guardianship was measured by applying technical guardians, such as antivirus software, and nontechnical/social guardians, such as living arrangement and asking for help. Technical guardianship was assessed by dichotomous answer categories to the following statements: “Cover your webcam on your computer or laptop;” “Use identity theft protection monitoring;” “Freeze your credit when you do not plan to use;” and “Use virus software and/or firewall on your computer.” Similarly, asking for help was assessed on a dichotomous scale: “If you witnessed one or more of the above scenarios, who did you ask for help?” where participants could check multiple answers (Table 2). We conceptualize living arrangement as capable guardianship. Studies refer to marriage and children as informal guardians that can protect individuals from victimization (Lee and Geistfeld, 1999; Langenderfer and Shimp, 2001; Fenge and Lee, 2018; Kennedy et al., 2021). Following previous studies (DeLiema, 2018), we assumed that adult relatives (e.g., partners and grown-up children) could function as informal capable guardians. Table 2 shows details and breakdowns of independent variables.

### 4.5. Control variables

This study used age as a control variable. Studies vary about determining the age of “older adults” when it comes to victimization. Some employed 50 (Lichtenberg et al., 2013, 2016), 55 (Federal Trade Commission, 2003; Pak and Shadel, 2011), 60 (Reisig and Holtfreter, 2013; Holtfreter et al., 2014), and 65 (Titus et al., 1995; AARP, 1999; Anderson, 2004, 2007, 2013; Holtfreter et al., 2006; Harrell and Langton, 2013; Harrell, 2015; Burnes et al., 2017; DeLiema, 2018; Fenge and Lee, 2018) as age cutoffs. We applied the age of 55 as a dividend between “younger” and “older” adults (<55 and > =55), hoping that more items in the sample of “older” adults will provide more generalizability. In addition, the computer became an integrated part of education in the mid-1980s, while the internet became a tool of mass communication not earlier than the mid-1990s (Molnar, 1997; Parker and Davey, 2014). Thus, people over 55 have not yet had computers and the internet as part of their everyday lives while growing up. This creates a generational gap between younger and older adults (Prensky, 2001). Younger adults have used computers and the internet as a necessity. In contrast, older individuals had to attain digital literacy later in life, and consequently, they might have more difficulties detecting online scams.

Victims under 55 comprised 35.3% (*n* = 915), and victims 55 and older made up 12.9% (*n* = 334) of the total sample (*n* = 2,589). Older victims generally attained higher education levels than younger ones: 58.4% of older victims had some college or college degrees, compared to 48.8% of younger victims. Employment differences turned out in the expected direction, with 55.96% of younger and 22.5% of older victims having full-time jobs, and 58.1% of older victims and 2.19% of younger victims being retired. Younger victims applied technical guardians at a higher rate than older victims, except for antivirus/firewall, which older victims applied at a higher rate (79.8%) than younger victims (65.9%). Computer familiarity showed a diverse picture, with a higher proportion of older victims having confidence in their basic computer skills (surfing and fixing some problems) than younger victims. However, younger victims had more confidence in programming (24.6%) than older ones (6.0%). Mean PC-time was higher (*M* = 30.6 h per week, *SD* = 27.3) for younger victims than older victims (*M* = 25.7 h per week, *SD* = 19.9). As expected, more older victims lived alone or with a partner, having no children around (79.6%) than younger victims (37.9%). For a between-group comparison of victim characteristics, see Table 3.

**Table 3:** Victim and non-victim demographics and routine activity theory measures by age group.

Victim characteristics	Victims 18–54 (*N* = 915)		Victims 55 and above (*N* = 334)	
Gender	*N*	Group %	*N*	Group %
Male	490	53.7%	197	59.2%
Female	409	44.8%	133	39.9%
LGBTQ+	12	1.3%	2	0.6%
No answer	4	0.4%	2	0.6%
Race (multiple answers)				
White	644	70.4%	299	89.5%
Black or African American	180	19.7%	11	3.3%
American Indian or Alaska Native	34	3.7%	6	4.5%
Asian	47	5.1%	14	1.8%
Native Hawaiian or Pacific Islander	13	1.4%	0	4.2%
Other	35	3.8%	9	2.7%
No answer	4	0.4%	0	0.0%
Education				
Less than a high school diploma	17	1.9%	2	0.6%
High school degree	194	21.2%	55	16.5%
Some college	186	20.4%	78	23.4%
A college degree	260	28.4%	117	35.0%
A master’s degree or higher	257	28.1%	82	24.6%
Employment				
In school	100	10.93%	3	0.9%
Full-time job	512	55.96%	75	22.5%
Part-time job	200	21.86%	39	11.7%
Unemployed	204	22.29%	31	9.3%
Retired	20	2.19%	194	58.1%
RAT – Computer familiarity				
Uncomfortable	118	12.9%	27	8.1%
Surf the net	181	19.8%	113	33.8%
Fix some problems	220	24.0%	117	35.0%
Fix most of the problems	171	18.7%	57	17.1%
Programming	225	24.6%	20	6.0%
RAT – PC-time				
Mean	30.6		25.7	
Median	21		21	
SD	27.3		19.9	
Min	0		0	
Max	246		129	
RAT – Technical Guardian				
Cover the web camera	605	66.5%	107	32.2%
Identity theft protection	481	53.3%	158	47.6%
Freeze credit card	413	45.9%	83	25.0%
Antivirus/Firewall	595	65.9%	265	79.8%
RAT – Living arrangement				
Living alone	138	15.1%	87	26.0%
Living with partner no children	208	22.8%	179	53.6%
Single parent	78	8.6%	6	1.8%
Living with partner and children	365	40.0%	38	11.4%
Living with parents	75	8.2%	4	1.2%
Other family type	40	4.4%	19	5.7%
Other arrangements	8	0.9%	1	0.3%
Victimization	N	% of age group	*N*	% of age group
1. Company impersonation	549	31.7%	116	14.0%
2. Tech support scam	485	28.0%	104	12.6%
3. Grandparent scam	396	22.9%	51	6.2%
4. Personal data verification	602	34.8%	225	27.2%
5. Advance fee fraud	426	24.6%	67	8.1%
6. Romance scam	401	23.2%	39	4.7%
Total (any of the above)	915	52.8%	334	40.4%

## 5. Findings

We created the “total victimization” variable considering the six fraud schemes included in the analysis. Respondents who answered “yes” to 1–6 scam scenarios were “victims” (1–6), and only if answered “no” to all the scams were “non-victims” (0). This variable forms the basis for the victim/non-victim groupings (e.g., older victims: any person 55 years of age and above who has been a victim of at least one of six scams; Table 3). Corroborating previous research results, the data suggest that older individuals are being victimized by any online fraud at a lower rate (40.4%) than younger ones (52.8%), with personal data verification, company impersonation, tech support scam, advance fee fraud, romance scam, and grandparent scam being the most frequent in rank for younger victims. The rank is similar for older victims, with grandparent scams being a little more frequent than romance scams. Despite having slightly different ranks, the prevalence of scams in the older victim group does not show a remarkable divergence compared to younger victims.

We hypothesized that older victims would differ in characteristics from younger victims*: H1 – Older individuals with more computer-intensive daily routines and who are more familiar with computers will be more likely victims of online fraud than younger adults.* To test this hypothesis, we created the PC-time and the computer familiarity variables so that the original interval scales were converted into ordinal level scales by the method of visual binning. Next, we merged the PC-time and the computer familiarity variables into a single new variable (RAT variable), an ordinal scale ranging from 2 to 10 (Table 1). As it is shown in Table 1, those who fell victim to any online scams were more familiar with computers than non-victims, both in the young (*M* rank_victims_: 1410.47, *M* rank_non-victims_: 1306.97) and the older group [*M* rank_victims_: 1169.2; *M* rank_non-victims_: 1062.64; H(3) = 84.666; *p* < 0.001]. The RAT scale (the merged computer familiarity and PC-time variable) showed a similar pattern: young people were more likely victimized than older adults, but victims, young and old, ranked higher in the RAT scale (young victims: 1270.19; old victims: 1081.59) than non-victims [young non-victims: 1192.42; old non-victims: 934.44; H(3) = 83.535; *p* < 0.001]. Therefore, the numbers corresponded with our first hypothesis.

Further analyzing the data, when it came to applying technical guardians (merged 0–4 scale variable from covering webcam, identity theft monitoring, freezing credit cards when not planned to use, and antivirus software/firewall), the victim groups ranged higher than the non-victim groups, independent of age [H(3) = 200.834; *p* < 0.001]. This means that victims applied more technical guardians than non-victims. However, younger victims were significantly more likely to put technical guardians in place as a result of previous victimization (*M* rank_yv_: 685.68) than older victims (*M* rank_ov_: 458.77; U = 97285.5; *p* < 0.01; Table 1). This detail refined our understanding of how much technical defensive techniques (or capable guardians) age groups tend to apply in order to fend off revictimization.

*H2 – We further assumed that those older individuals who live alone, without a partner or children, are more likely will be victimized than those who live in different living arrangements.* To test this assumption, we used the total victimization variable (see Table 3), then we made pairwise comparisons between living arrangements within the older victim group with Mann–Whitney U tests. Contrary to the expectations, those who were single parents were significantly less likely to be victimized than those who lived in any other living arrangements (U = 8851.000; *p* = 0.033), including those living with a partner and children (U = 839.000; *p* = 0.016; Table 4).

**Table 4:** Employment type, and nontechnical guardians (reporting/asking for help, living arrangement) by victim age groups.

Employment and victimization; Older individuals – Fisher’s Exact test (*n* = 667)				
Victimization of age group 55+	Group %		Fisher *p*	
	Retired (*n* = 493)	Full time job (*n* = 174)		
1.Company impersonation	12.60%	19.50%	0.032	
2. Tech support scam	13.00%	15.50%	N.S.	
3. Grandparent scam	4.30%	10.30%	0.007	
4. Personal data verification	25.80%	31.60%	N.S.	
5. Advance fee fraud	5.70%	13.20%	0.002	
6. Romance scam	2.40%	11.50%	0.001	
Computer-related job and victimization; Younger and older individuals – Fisher’s Exact test (*n* = 1,051)				
Victimization of those with computer-related vocational daily routines	Group %		Fisher *p*	
	Victim group (55+)	Victim group (18–54)		
1.Company impersonation	18.9%	42.5%	0.001	
2. Tech support scam	14,6%	39.2%	0.001	
3. Grandparent scam	11.4%	33.4%	0.001	
4. Personal data verification	32.4%	43.2%	0.008	
5. Advance fee fraud	14.6%	34.3%	0.001	
6. Romance scam	10.8%	34.7%	0.001	
Asking for help/Reporting; Younger and older victims – Fisher’s Exact test (*n* = 1,249)				
Asking for help from whom?	Group %		Fisher *p*	
	Victim group (55+)	Victim group (18–54)		
The same people who contacted me	10.20%	14.10%	N.S.	
IT personnel or team	7.50%	18.40%	0.001	
Civic organizations	2.70%	7.70%	0.001	
Federal Trade Commission	6.60%	7.20%	N.S.	
Lawyer	1.80%	8.00%	0.001	
Family Member	8.40%	12.80%	0.027	
Living Facility Administrator	1.20%	3.30%	0.034	
Other	13.20%	2.10%	0.001	
No one	64.10%	14.00%	0.001	
Living arrangement and victimization - Pairwise comparisons with Mann–Whitney test				
Living arrangement (*N* = 826)	Mean Ranks		Mann–Whitney U-stat	*p*
Living alone vs. all other 55+	404.72	416.63	-	N.S.
Living w partner no children vs. all other 55+	421.53	405.11	-	N.S.
Single parent vs. all other 55+	330.61	416.41	8851.000	0.033
Living w partner, children vs. all other 55+	446.04	409.91	-	N.S.
Other family arr. vs. all other 55+	397.17	414.69	-	N.S.
Living w partner no children vs. living w partner, children	249.99	265.43	-	N.S.
Other family arr. vs. living w partner, children	64.8	72.71	-	N.S.
Single parent vs. Living w partner, children	44.46	59.27	839.000	0.016

*H3 – Older victims are less likely to report and ask for help than younger victims.* When it comes to asking for help or reporting, older victims (64.10%) were indeed significantly more likely than younger ones (14.00%) to have said that they did not reach out to anyone (*p* = 0.001). Apart from reporting to the Federal Trade Commission or to the same person who contacted the victim, all report options showed statistically significant differences per age group: younger victims were more likely than older victims to report to the IT personnel or team (*p* = 0.001), to a civic organization (*p* = 0.001), a lawyer (*p* = 0.001), a family member (*p* = 0.027), and a living facility administrator (*p* = 0.034; Table 4).

**Figure 2:**
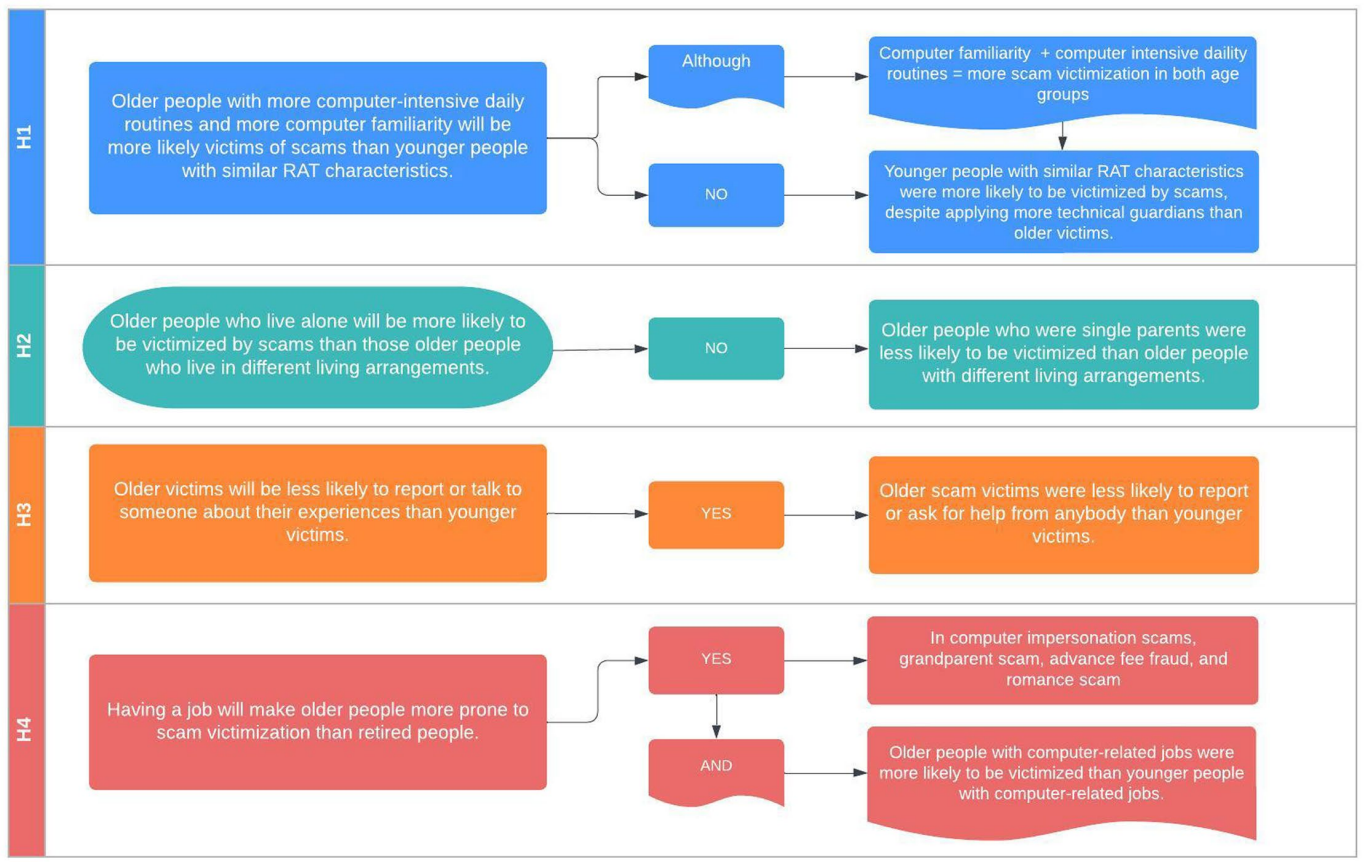
Results graphic.

*H4 – Having a job makes older people more prone to online victimization than retired individuals.* First, we compared full-time employee and retired victims within the older victim group. According to the results, having a full-time job indeed made older victims more prone to online fraud victimization when it comes to company impersonation scams (Fisher *p* = 0.032), grandparent scams (Fisher *p* = 0.007), advance fee fraud (Fisher *p* = 0.002), and romance scams (Fisher *p* = 0.001). The difference between full-time employed and retired older victims was most remarkable in advance fee fraud and romance scams. No statistically significant relations were revealed in tech support and personal data verification scams (Table 4). In a second step, we wanted to see specifically whether older individuals with computer-related job activities are more likely to be victimized than younger individuals with computer-related jobs. This time, we measured age-group differences of all participants with a full-time or part-time job who indicated at least an hour of computer use while at work (*n* = 1,051). The Fisher test showed a statistically significant relationship between using a computer while at work and being victimized for all types of online fraud. However, younger individuals with computer-related vocational routines were significantly more likely to be victimized by any scam than older individuals (Table 4; Figure 2).

## 6. Discussion

Despite its prevalence and relatively serious consequences, fraud against older people has not yet been studied extensively in a social context. The works tend to focus on financial exploitation of older adults by caretakers, but financial fraud by strangers is often missed out from the analysis. In addition, studies do not include cases where perpetrators only interacted with victims online or by phone (Burnes et al., 2017; DeLiema, 2018). Furthermore, the role of social context variables (demographics, living arrangements, job market activity) in online fraud victimization has not been revealed yet, because of the lack of available data on victims’ sociodemographic characteristics (DeLiema, 2018; Whitty, 2019). The present study aims to fill these above gaps by looking at situational and demographic characteristics of older fraud victims to investigate what makes older adults vulnerable to online scams and what the potential protective factors are. The current study’s novelty is that, by focusing on demographics and cyber victimization factors, it reveals unique findings of risk and protective factors for older victims, compared to younger victims, utilizing routine activity theory (Cohen and Felson, 1979). The paper test routine activities theory to compare the differences in online fraud vulnerabilities of younger victims aged 18–54 and older victims of 55 and above. In addition to computer time and computer familiarity, we included independent variables such as living arrangement, employment, and reporting fraud or asking for assistance in the analysis. We assumed that older fraud victims differ in situational and sociodemographic characteristics from younger fraud victims. We investigated the differences that function as risk or protective factors utilizing non-parametric statistical tests such as Mann–Whitney U and Kruskal-Wallis H tests.

Our hypotheses were partly supported. People with more computer familiarity and computer-bound daily routines fell victims of scams on a greater level in both age groups, however, younger computer savvy individuals fell victim to scam scenarios still more likely than older ones with similar characteristics. In some cases (personal data verification and total victimization), the difference between the age groups was smaller. However, the difference was greater in company impersonation, grandparent scam, advance fee fraud, and romance scam. Interestingly, other than romance scam, which typically starts by contacting the targets on online social media or dating websites (Cross, 2020b), all scam types where older people had a higher chance of victimization typically started with a phone call from the scammers (The scam scenarios in the survey described how the scammer made the initial contact). This indicates that older people are more approachable by phone than younger people, perhaps because of their relatively low level of online presence compared to younger people. Holtfreter et al. (2014) also found that the telephone was the most common contact method for older people targeted for consumer fraud. On the other hand, this finding suggests that when approached via phone, older people were more likely than younger ones to believe the scammers, compared to email-initiated frauds (personal data verification scam and advance fee fraud in our survey) where the level of risk to be victimized was not so different in young and older adults.

Victims reported a higher computer skills level than non-victims in both age groups. However, older victims self-reported a significantly higher computer savviness than non-victims. To be precise, older people who fell victim to scams were less likely to be computer savvy than any young respondent, but more so than old non-victims. Perhaps cognitive biases such as overconfidence in computer skills (González et al., 2015) and the illusion of control (Thompson, 1999; Matute et al., 2015) are some reason older victims are more reluctant to apply simple technical guardians such as a webcam cover, identity theft monitoring, and credit card freeze, compared to younger victims, even after being scammed. This difference between young and old victims comes to light only after comparing the victim age groups since, in general, the older group reported a higher level of basic computer familiarity (surfing the net and fixing minor computer problems) than the younger group. In addition, although older respondents’ computer familiarity was lower than younger respondents’ in computer programming, that is not the skillset one would need to recognize potential scams. Instead, people need more awareness of how scammers typically approach targets and perhaps more technical guardians to prevent victimization. However, the data corresponds that older victims not only tend to overestimate their computer skills but are also less likely to install essential technical guardians, even after being scammed than younger victims. The result was similar when we included PC-time (time spent on a computer daily) into the analysis: older victims’ mean average time on a computer lags behind that of any younger respondents (victims and non-victims) but exceeds older non-victims’ computer time. Thus, spending time on a computer, being (over)confident in computer skills, and lacking technical guardians all increase the risk of online fraud victimization for everyone, but it makes seniors more suitable targets than younger people.

We further conceptualized living arrangement and asking for help/reporting fraud as nontechnical guardians. Research indicates that loneliness and isolation make individuals more willing to answer unknown phone calls (Langenderfer and Shimp, 2001), and the presence of relatives and family members can function as an extra pair of eyes/ears that can prevent scams (DeLiema, 2018). In the current sample, however, quite unexpectedly, older people who were single parents significantly less likely fell victim to scams than older victims in other living arrangements. In addition, older single parents were significantly less likely to experience scams than older people with young children *and* a partner around. The question applies then, why does being a single older parent means protection against scams?

To better understand the context of single parenthood, we borrow the concept of “grandparenthood” (Dolbin-MacNab, 2019), according to which relative caregivers, such as grandparents, can serve as key sources of support within families. As society is aging, older people are more likely to serve as sole caretakers or guardians of grandchildren whose parents are perished, incarcerated, struggling with addiction or mental health issues, or where the parent is young or inexperienced in childrearing (Saxena and Brotherson, 2021). Other reasons for grandparenthood include unstable home life or parents’ homelessness, lack of financial resources or general ability, domestic violence in the home, divorce, or military deployment (Hayslip and Kaminski, 2005). Research shows that custodial grandparents, adults who are caring for their grandchildren full-time, are becoming more prevalent. In 2000, 5.7 million grandparents lived with their grandchildren (Hayslip and Kaminski, 2005), and approximately 2.4 million individuals raised their grandchildren in the United States. In 2014, approximately 7.8 million (one in 10) children lived with 4.9 million grandparents (Generations United, 2014). Although we do not have information about the exact circumstances of single parenthood in our sample, we propose that “grandparenthood” can be a feasible explanation of less scam victimization. It is realistic that people above 55, who are sole guardians of young children, are extremely cautious not to enter any risky situations, including answering cold calls or jumping into risky winning schemes. They might intend to spend their income very carefully, especially if they are retired and live on a fixed income. Thus, single parenthood and fiscal shortages might function as capable guardians from financial fraud for older adults. However, future research must look into the details and context of how living arrangements can serve as a risk or protective factor in online scamming in the context of RAT.

Asking for help is never an easy task, but, according to our results, for older victims, it can be even more challenging than for younger ones. The results suggest that younger victims are significantly more likely to ask for help from anyone, except for reporting to the Federal Trade Commission, an agency accepting scam reports and quite well known by the American people. In contrast, older victims typically do not talk to anyone or report anywhere their scam experiences. Curiously enough, family members were the least likely contacted by older victims among the options offered (lawyer, IT personnel, civic organization, living facility administrator, etc.). Unfortunately, our study did not ask *why* victims do not confide in family members and relatives. These questions are highly recommended to investigate in future research. Asking for help/reporting victimization could help reduce repeated victimization, thus being an essential prevention tool, and research should look at what role reporting (or the lack of it) plays in re-victimization of older people.

An additional characteristic of older victims is the type of employment. In our sample, older victims with full-time jobs were significantly more likely to experience company impersonation, grandparent scam, romance scam, and advance fee fraud than retired victims with no vocational routine activities. Although the scam scenarios we provided in the survey indicated that these same scams tend to begin with a phone call, most scams operate on a mixed utilization of phone and computer-based communication. Hence, after the initial telephone call, the target might be asked to go to the computer and continue communicating electronically, and a full-time job might provide more proximity to a computer. On the other hand, full-time employees’ higher level of victimization might have to do with more confidence in spending money. This finding is in line with previous research suggesting that being confident in finances and having rich financial literacy skills are indeed risks of online fraud victimization (Fenge and Lee, 2018). Another possible explanation for the relatively more vulnerability of full-time workers in the older age group might be a false sense of security provided by the employer’s computer system, not realizing that fraudsters tend to apply social engineering techniques that cannot be entirely prevented by technical guardians such as firewall or antivirus software. This detail is worth the attention, even though computer-related vocational routines more likely turned into risky daily lifestyle routines for younger than older individuals in this sample.

### 6.1. Limitations

In the current study, it was impossible to have an exact measure of capable guardians and online lifestyle/routine activities. In addition, content validity regarding computer familiarity, a self-assessment measure can be another concern. Perhaps it would be more adequate to measure computer skills by providing detailed descriptions. Furthermore, firewall and antivirus programs are nowadays included in modern computer operating systems; thus, measuring capable guardianship could be more valid by applying a detailed list of modern computer security programs that users can select.

The list of online manipulation schemes applied in the study is not complete. Online fraud constantly evolves, especially when offline communication is restricted due to natural emergencies such as the COVID-19 pandemic. Daily routine activities increasingly shift to online platforms as digital connectedness grows. Due to demographic, public health characteristics and relatively new connectedness, older adults represent an especially vulnerable age group. In addition, as older adults’ online presence grows, the range of online manipulations is expected to emerge and expand (Cross, 2020a). Prevention programs and policies can never be comprehensive to tackle all versions of online manipulations. Still, research can help map the risks by providing more precise scales to study online victimization. Updating surveys regarding new online manipulation schemes is imperative to get a fuller picture of older adult victimization. In the future, it may be necessary to implement face-to-face interviewing to provide more opportunities for participants to share circumstances of victimization.

Furthermore, survey items must be designed in consultation with older adult victims of financial fraud in order to achieve measurement accuracy. Future elder fraud victimization studies should even include close proxy respondents (caretakers) to represent older adults with cognitive impairment that limit direct participation. Research suggests (Cross, 2016) that there is no common method of scamming, and thus, there is no universal explanation for fraud victimization. Instead, financial fraud can take many forms, and it is essential to apply case-based investigation. Since individuals with specific socio-economic backgrounds can be targets of different fraud types, each type of financial fraud should be studied individually.

Because of sample and data characteristics (Kitchen, 2009; Nahm, 2016), we utilized Mann–Whitney U tests to compare differences between the age groups considering victimization. However, this statistical test has its weaknesses. For instance, it requires lengthy calculations and is prone to human error. Furthermore, it does not explain which variable causes the difference between the groups (Nachar, 2008). Future research must be aware of these weaknesses and apply more appropriate samples and statistical tests to inspect group differences. The use of multilevel modeling in future analyses is, as well, would be recommended, to identify data hierarchies and residual components.

### 6.2. Practical and theoretical implications

The results suggest that older victims are more reluctant than younger ones to ask for help. Moreover, relatives and family members are the least likely to know about their loved ones’ victimization. Therefore, close proxy individuals who take care of older adults should also be the focus of awareness-raising campaigns to learn more about early signs of financial fraud in order to avoid scams and help victims overcome their losses.

Older adults with full cognitive capacity may still be in the workforce, but they exhibit risk factors unique to them, such as better financial situations (savings and assets), more confidence in handling large investments, and lacking formal and informal capable guardians (i.e., not applying technical tools, not having somebody in close proxy to confide in). Employers should address the unique vulnerabilities of the aging workforce by providing age-appropriate financial fraud prevention programs tailored to their needs. Perhaps it is time for employers to realize that technical guardians are not meant to protect workers fully. Instead, sophisticated online frauds psychologically manipulate victims to give out their valued assets. Awareness programs must cover social engineering techniques, how con artists target and manipulate victims, and make it clear that it can happen to everyone, regardless of age. Realizing the everyday nature of scams can provide confidence to victims to report or ask for help so that scams will likely be avoidable for them in the future.

### 6.3. Future research directions

The current study tested online fraud victimization from a sociodemographic and routine activities’ perspective; however, it did not include victims’ personal characteristics in the analysis. The mixed results of the study suggest that online and telephone fraud victimization is a more complex issue and underlying measures must be responsible for victimization and protection. Therefore, the role of sociodemographic characteristics and personal measures should be further studied in the scam victimization of older ages. For example, Whitty (2019) suggests that impulsivity and self-control measures should be part of the analysis because victims tend to act out of urgency and sensation -- in seeking a job opportunity, a lucrative investment, or an item to purchase. The effects of personal characteristics on victimization can be amplified due to the pandemic, when the recent loss of close relatives, sudden unemployment, or other kinds of financial hardship impacts common sense of careful judgment. Furthermore, additional factors such as age-related changes in cognition may mediate scam vulnerability of even older age groups (Boyle et al., 2012). Future studies must examine how these factors, combined with individuals’ daily routines and missing capable guardians, influence scam vulnerabilities of older people. The risk and protective factors should be also examined separately in younger and older retired individuals, so that characteristics of victims 65 and above can be identified. Although there is a staggering number of scam victimization reported (FBI, 2020), researchers know less about unreported scams that may or may not result in money loss, but likely inflicts emotional struggle, isolation of victims, and estrangement from family and community. Future research must investigate what role reporting (or the lack of it) plays in repeat victimization of scam victims.

Online fraud constantly evolves, with fraudsters inventing new subtle manipulation techniques. Although the current study involved six of the most common online fraud scenarios, victims’ characteristics should be further tested for vulnerability to scams operating with new social engineering methods. The risk factors deriving from computer-related vocational activities and single parenthood of older age groups, and the possible protective roles of close proxy family members and caretakers must be further studied in light of the current findings.

## Data availability statement

The datasets presented in this study can be found in online repositories. The names of the repository/repositories and accession number(s) can be found at: Longitudinal Survey of Cybercriminology: https://data.lib.vt.edu/articles/dataset/Longitudinal_Survey_of_Cybercriminology_November_2019/17092283.

## Ethics statement

The studies involving human participants were reviewed and approved by Virginia Tech Institutional Review Board #19-1010. The patients/participants provided their written informed consent to participate in this study.

## Author contributions

The author confirms being the sole contributor of this work and has approved it for publication.

## Funding

The research was funded by the Institute for Society Culture and Environment at Virginia Tech, and the Niles Grant at the College of Arts and Liberal Sciences at Virginia Tech.

## Conflict of interest

The author declares that the research was conducted in the absence of any commercial or financial relationships that could be construed as a potential conflict of interest.

## Publisher’s note

All claims expressed in this article are solely those of the authors and do not necessarily represent those of their affiliated organizations, or those of the publisher, the editors and the reviewers. Any product that may be evaluated in this article, or claim that may be made by its manufacturer, is not guaranteed or endorsed by the publisher.
